# Protective effect of pre- and post-vitamin C treatments on UVB-irradiation-induced skin damage

**DOI:** 10.1038/s41598-018-34530-4

**Published:** 2018-11-01

**Authors:** Saki Kawashima, Tomoko Funakoshi, Yasunori Sato, Norikatsu Saito, Hajime Ohsawa, Katsumi Kurita, Kisaburo Nagata, Masayuki Yoshida, Akihito Ishigami

**Affiliations:** 1 Molecular Regulation of Aging, Tokyo Metropolitan Institute of Gerontology, Tokyo, 173-0015 Japan; 2 Department of Life Science and Bioethics, Graduate School of Medicine, Tokyo Medical and Dental University, Tokyo, 113-8510 Japan; 3 Department of Biomolecular Science, Faculty of Science, Toho University, Chiba, 274-8510 Japan; 4 Department of Bioenvironmental Pharmacy, Faculty of Pharmaceutical Sciences, Hokuriku University, Ishikawa, 920-1181 Japan; 5 Risou Co., Ltd, Tokyo, 104-0061 Japan

## Abstract

Several studies have reported the effects of vitamin C (L-ascorbic acid, AA) on ultraviolet B (UVB)-induced cell damage using cultured keratinocytes. However, the epidermis consists of multiple cell layers, and the effect of AA on UVB-induced damage to the human epidermis remains unclear. Therefore, we investigated the effect of AA on UVB-induced skin damage using reconstituted human epidermis. The reconstituted human epidermal surface was treated with 100 and 500 mM AA and cultured for 3 h before (pre-AA treatment) or after (post-AA treatment) 120 mJ/cm^2^ UVB irradiation. Pre- and post-AA treatments of the epidermal surface suppressed UVB-induced cell death, apoptosis, DNA damage, reactive oxygen species (ROS) production, and the inflammatory response by downregulating tumour necrosis factor-α (TNF-α) expression and release. Moreover, the pre-AA treatment was more effective at preventing UVB-induced skin damage than the post-AA treatment. In summary, pre- and post-AA treatments of the epidermis prevent UVB-induced damage.

## Introduction

Solar ultraviolet (UV) radiation is the most important environmental factor causing cancer and photoaging, such as wrinkle formation, acute erythema, and tanning of human skin^1–3^. UV radiation is divided into three wavelength bands: UVA (320–400 nm), UVB (280–320 nm), and UVC (100–280 nm). UVC is absorbed by the ozone layer in the atmosphere. Approximately 90 to 95% of UVA and 5 to 10% of UVB radiation reaches the ground^1,4^. UVB is absorbed by the epidermis and induces DNA damage by forming cyclobutane pyrimidine dimers and pyrimidine (6–4) pyrimidone photoproducts^1,4^. Moreover, UVB reacts with water in the skin to produce reactive oxygen species (ROS), including singlet oxygen, superoxide anions, and hydroxyl radicals^5–7^. UVB-induced ROS react with lipids, proteins, and DNA. UVB-induced oxidative damage leads to apoptosis and gene mutations^2–4^. UVB also induces inflammation by promoting pro-inflammatory cytokine production and release from cells^8,9^. Epidermal keratinocytes express several antioxidant enzymes, such as superoxide dismutase (SOD), catalase (CAT), glutathione-S-transferase (GST), and glutathione peroxidase (GSH-Px), which contribute to maintaining the cellular redox balance by scavenging excess ROS^10^. However, UVB decreases antioxidant enzyme activity and increases intracellular ROS levels in the epidermis, thus disrupting the cellular antioxidant defence system^11–13^. The accumulation of ROS-mediated skin damage leads to carcinogenesis and skin ageing^2,3^. Therefore, endogenous antioxidants are required to protect against UVB-induced skin damage.

Vitamin C (L-ascorbic acid, AA) acts as an electron donor and reduces the production of ROS such as superoxide radicals^14^, hydroxyl radicals^15^, and singlet oxygen^16^. In guinea pigs and primates, including humans, the ability to synthesize AA has been lost due to many mutations in the L-gulonolactone oxidase gene, whereas most vertebrates can synthesize AA *in vivo*^17^. AA reduces UVB-induced ROS production and prevents ROS-mediated cell death and oncogenesis in epidermal keratinocytes^18,19^. Additionally, AA suppresses UVB-induced inflammation by downregulating cytokine production^20^. Several studies have reported the effects of AA on UVB-induced cell damage using cultured keratinocytes, although the epidermis actually consists of multiple cell layers.

Previously, we generated senescence marker protein-30 (SMP30) knockout hairless mice, a strain of mice that, like humans, cannot synthesize AA *in vivo*^21^. SMP30 is an essential enzyme in the AA biosynthetic pathway^17^. Using this SMP30 knockout hairless mouse model, we previously reported that AA deficiency led to epidermal atrophy and the development of excessive skin pigmentation in AA-insufficient SMP30 knockout hairless mice following exposure to UVB irradiation^21^. However, the effect of AA on UVB-induced damage to the human epidermis remains unclear. Therefore, in this study, we investigated the effect of AA on UVB-induced skin damage using the reconstituted human epidermis.

## Results

### AA uptake into the reconstituted human epidermis

**Figure 1:**
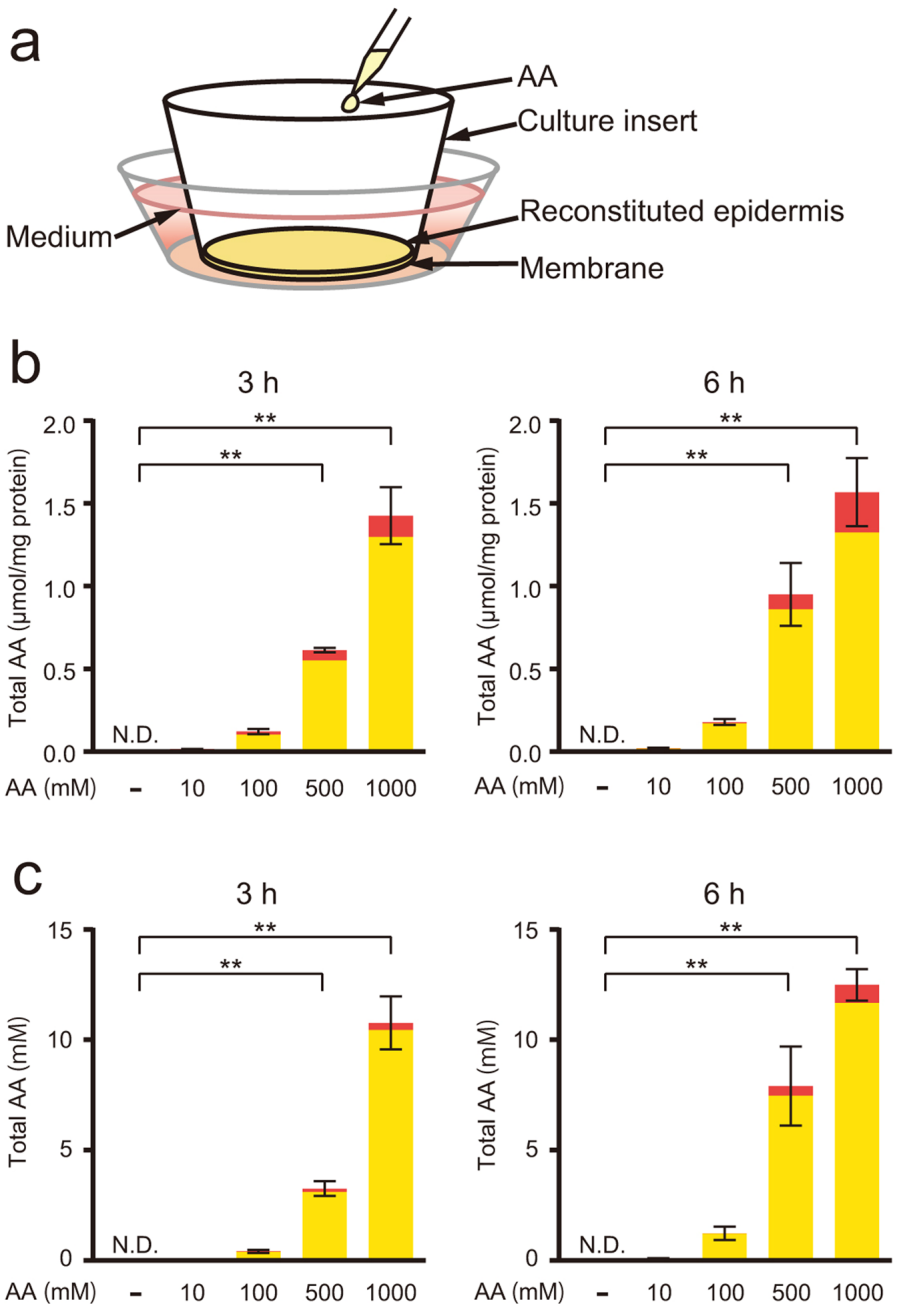
AA uptake in the reconstituted human epidermis. (**a**) Schematic representation of the cultured reconstituted human epidermis. The epidermis was constructed on the membrane of the culture insert. (**b** and **c**) Treatments with 10, 100, 500, and 1000 mM AA and water as a control were applied to the reconstituted human epidermal surface and cultured for 3 and 6 h. Total AA levels, including AA (yellow column) and DHA (red column), in the (**b**) epidermis and the (**c**) medium under the membrane were measured as described in the Methods. AA and DHA levels in the epidermis were normalized to the protein content in the epidermis. Values are presented as the means ± SEM of three wells. The statistical analysis was performed using one-way ANOVA followed by Dunnett’s *post hoc* test. **P < 0.01. N.D., not detected.

We applied 10, 100, 500, and 1000 mM AA, concentrations that are equivalent to 0.2, 2.0, 9.9, and 20% of AA, respectively, to the epidermal surface to confirm AA uptake into the reconstituted human epidermis (Fig. 1a). The epidermis was cultured for 3 and 6 h, and then the levels of AA and dehydroascorbic acid (DHA) in the epidermis and the medium under the membrane were measured (Fig. 1b,c). AA was detected in the epidermis when 10, 100, 500, and 1000 mM AA were applied for 3 and 6 h, but it was not detected in samples that were not treated with AA (Fig. 1b). Total AA levels in the epidermis were 8-, 38-, and 89-fold higher when 100, 500, and 1000 mM AA were applied for 3 h, respectively, than when 10 mM AA was applied. Additionally, total AA levels in the epidermis were 9-, 45-, and 75-fold higher when 100, 500, and 1000 mM AA were applied for 6 h, respectively, than when 10 mM AA was applied. Moreover, AA was detected in the medium under the membrane when 10, 100, 500, and 1000 mM AA were applied for 3 and 6 h, but it was not detected in media from samples that were not treated with AA (Fig. 1c). Total AA levels in the medium were 9-, 67-, and 222-fold higher when 100, 500, and 1000 mM AA were applied for 3 h, respectively, than when 10 mM AA was applied. Moreover, total AA levels in the medium were 13-, 85-, and 134-fold higher when 100, 500, and 1000 mM AA were applied for 6 h, respectively, than when 10 mM AA was applied.

### UVB induced skin damage

**Figure 2:**
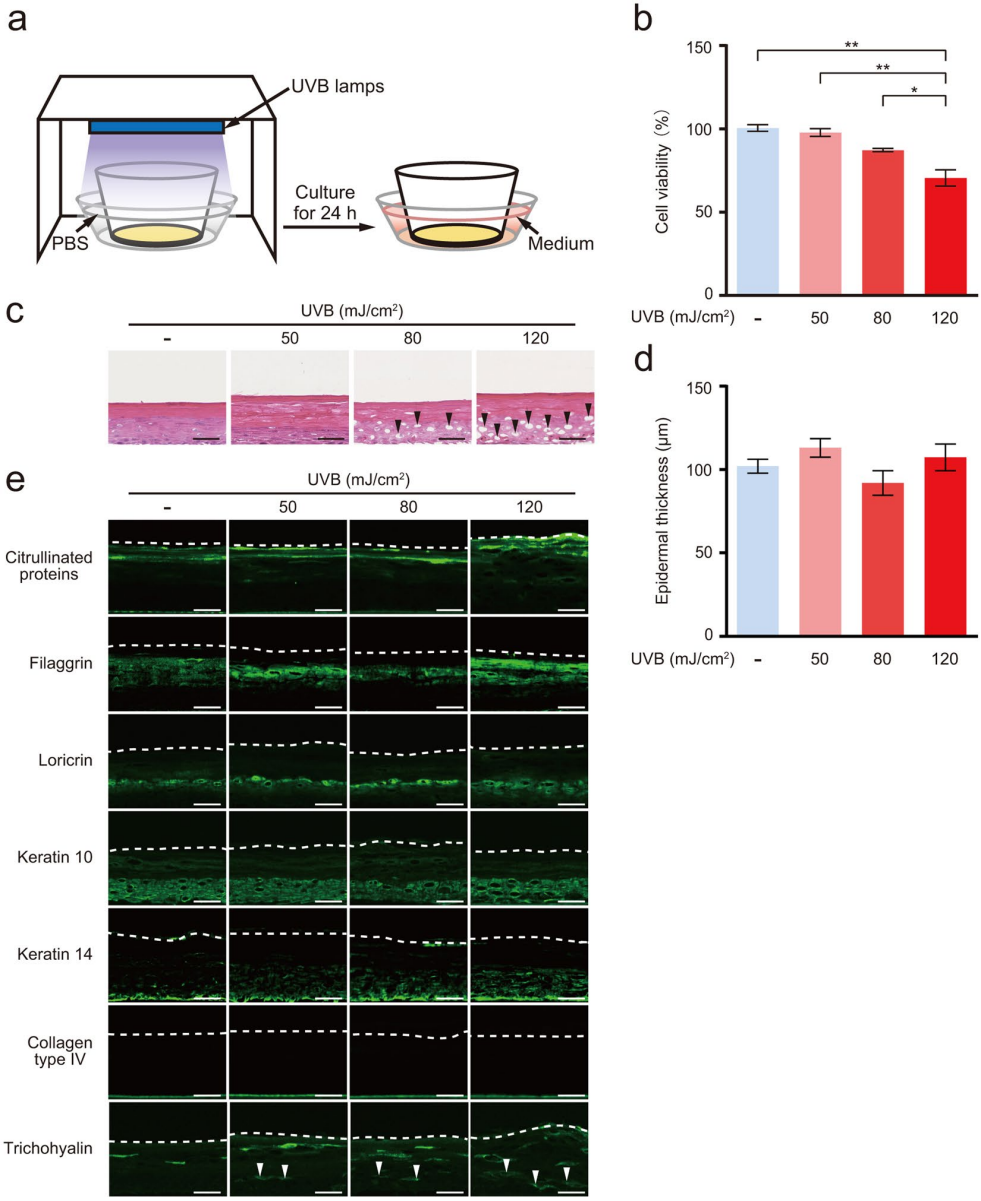
UVB induced cell damage in the reconstituted human epidermis. (**a**) The reconstituted human epidermis was irradiated with 50, 80 or 120 mJ/ mJ/cm^22^ UVB. For irradiation, the medium was replaced with PBS beneath the culture insert, and the culture plate was irradiated without the plastic plate lid. After irradiation, PBS was replaced with fresh medium, and the epidermis was cultured for 24 h. (**b**) The viability of cells in the UVB-irradiated epidermis was measured using the MTT assay. Values are presented as the means ± SEM of three wells. (**c**) Histological changes in the UVB-irradiated epidermis. Black arrowheads indicate gaps formed by holes. Bars = 50 µm. (**d**) Thickness of the UVB-irradiated epidermis. Epidermal thickness was measured as the distance between the top of the membrane and the bottom of the cornified layer in three randomly selected fields from each epidermis. Values are presented as the means ± SEM of three wells. (**e**) Immunofluorescence staining for citrullinated proteins, filaggrin, loricrin, keratin 10, keratin 14, collagen type IV, and trichohyalin in the UVB-irradiated epidermis. The dotted line represents the bottom of the cornified layer. White arrowheads indicate trichohyalin. Bar = 50 µm. The statistical analysis was performed using one-way ANOVA followed by Tukey’s *post hoc* test. *P < 0.05, **P < 0.01.

The reconstituted human epidermis was irradiated with 50, 80, or 120 mJ/cm^2^ UVB and cultured for 24 h (Fig. 2a). The viability of cells was measured using the methyl thiazolyl tetrazolium (MTT) assay. The cell viability of the 50, 80, and 120 mJ/cm^2^ UVB-irradiated epidermis was 97.3 ± 2.3, 86.8 ± 1.0, and 70.2 ± 4.9%, respectively (Fig. 2b). Cell viability of the 120 mJ/cm^2^ UVB-irradiated epidermis was significantly reduced by 29.8% compared with that of the non-irradiated epidermis. The histological analysis of the UVB-irradiated epidermis is shown in Fig. 2c. Many remarkable gaps formed by holes were observed in the basal, spinous, and granular layers of the 80 and 120 mJ/cm^2^ UVB-irradiated epidermis. No significant differences in epidermal thickness were observed among the 50, 80, and 120 mJ/cm^2^ UVB-irradiated epidermis and non-irradiated epidermis (Fig. 2d). Immunofluorescence staining for markers of terminally differentiated keratinocytes, such as citrullinated proteins and filaggrin, which is located in the cornified layer; loricrin, which is located in the granular layer; keratin 10; which is located in the spinous layer; keratin 14, which is located in the basal layer; and collagen type IV, which is located in the basement membrane, is shown in Fig. 2e. Citrullinated proteins, filaggrin, loricrin, keratin 10, keratin 14, and collagen type IV were detected in the appropriate layers of the epidermis in the 50, 80, and 120 mJ/cm^2^ UVB-irradiated and non-irradiated samples. An apparent change in localization of the citrullinated proteins, filaggrin, loricrin, keratin 10, keratin 14, and collagen type IV was not observed among the 50, 80, and 120 mJ/cm^2^ UVB-irradiated epidermis and non-irradiated epidermis. Moreover, significant differences in levels of the filaggrin, loricrin, keratin 10, keratin 14, and collagen type IV mRNAs were not observed among the 50, 80, and 120 mJ/cm^2^ UVB-irradiated epidermis and non-irradiated epidermis (Supplementary Fig. S1). Trichohyalin is located in the granular and cornified layers^22^, and it was detected in the granular and cornified layers of the non-irradiated epidermis (Fig. 2e). However, trichohyalin was detected in the spinous, granular, and cornified layers of the 50, 80, and 120 mJ/cm^2^ UVB-irradiated epidermis (Fig. 2e). In addition, levels of the trichohyalin mRNA in the 50, 80, and 120 mJ/cm^2^ UVB-irradiated epidermis were 4-, 6-, and 6-fold higher, respectively, than in the non-irradiated epidermis (Supplementary Fig. S1).

### UVB induced DNA damage and apoptosis

**Figure 3:**
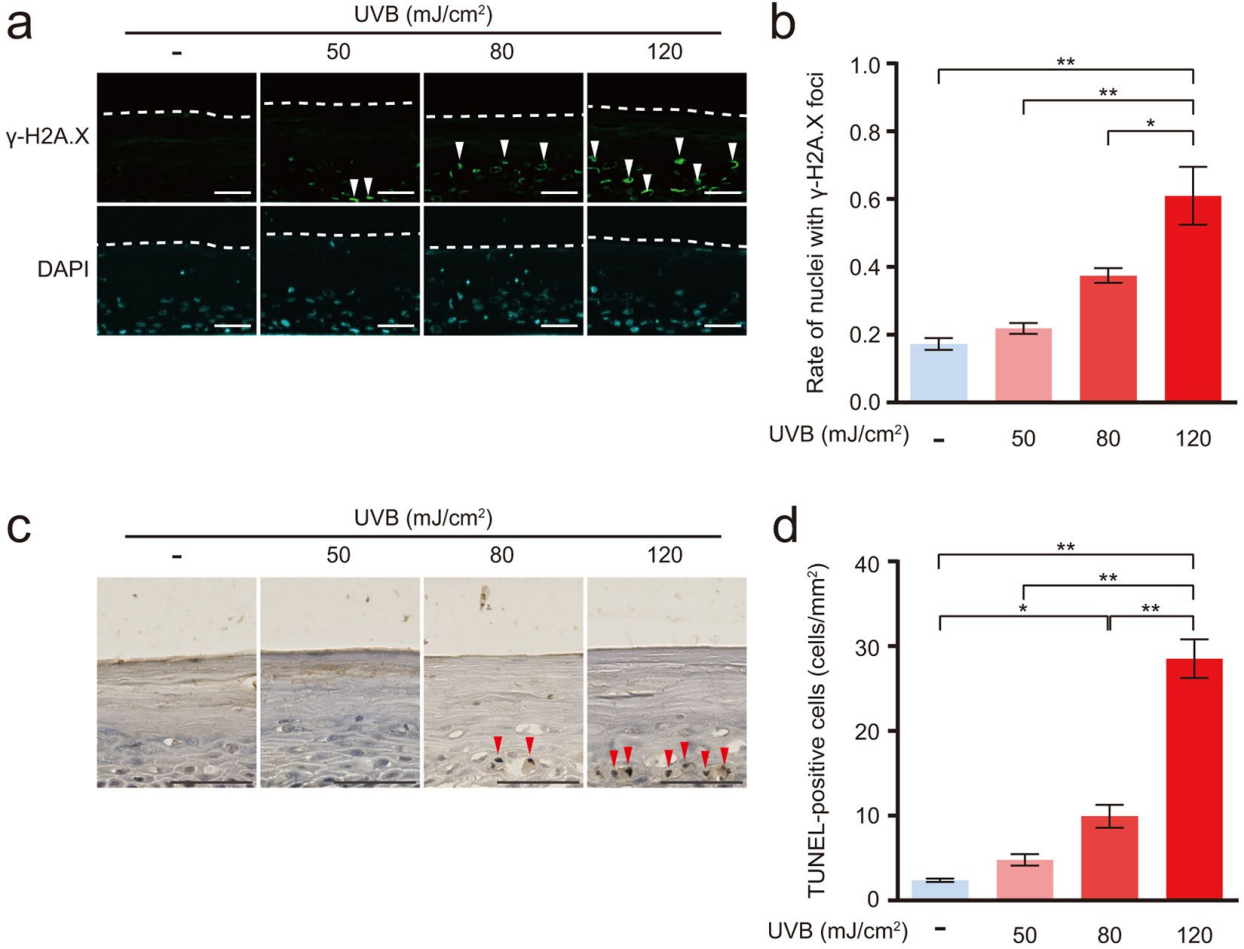
UVB induced DNA damage and apoptosis in the reconstituted human epidermis. The reconstituted human epidermis was irradiated with 50, 80 or 120 mJ/cm^2^ UVB. After UVB irradiation, the epidermis was cultured for 24 h. (**a**) Immunofluorescence staining for γ-H2A.X as a marker of DNA double-strand breaks. The dotted line represents the bottom of the cornified layer. White arrowheads indicate γ-H2A.X-positive nuclei. Bar = 50 µm. (**b**) The percentage of γ-H2A.X-positive nuclei per DAPI-positive nuclei. Values are presented as the means ± SEM of three wells. (**c**) Apoptotic cells were detected by TUNEL staining. Red arrowheads indicate TUNEL-positive cells. Bar = 50 µm. (**d**) Number of TUNEL-positive cells under the cornified layer. Values are presented as the means ± SEM of three wells. The statistical analysis was performed using one-way ANOVA followed by Tukey’s *post hoc* test. *P < 0.05, **P < 0.01.

Images of immunofluorescence staining for phospho-histone H2A.X (γ-H2A.X), a marker of DNA double strand breaks, are shown in Figure 3. γ-H2A.X-positive staining was observed in the nuclei of cells in the basal, spinous, and granular layers in the 50, 80, and 120 mJ/cm^2^ UVB-irradiated epidermis. The percentage of nuclei with γ-H2A.X foci in the 120 mJ/cm^2^ UVB-irradiated epidermis was a significantly increased by 4-fold compared with that in the non-irradiated epidermis (Fig. 3b). MTT assay is not a true cell viability assay, because it shows the mitochondrial dehydrogenase activity, we next determined apoptotic cells in UVB-irradiated epidermis. The number of apoptotic cells was assessed using the TUNEL assay. TUNEL-positive cells displaying brown-stained nuclei were detected in the basal and spinous layers of the 80 and 120 mJ/cm^2^ UVB-irradiated epidermis (Fig. 3c). The number of TUNEL-positive cells in the 80 and 120 mJ/cm^2^ UVB-irradiated epidermis was significantly increased by 4- and 12-fold, respectively, compared with that in the non-irradiated epidermis (Fig. 3d).

### Effect of AA on UVB-induced cell death

**Figure 4:**
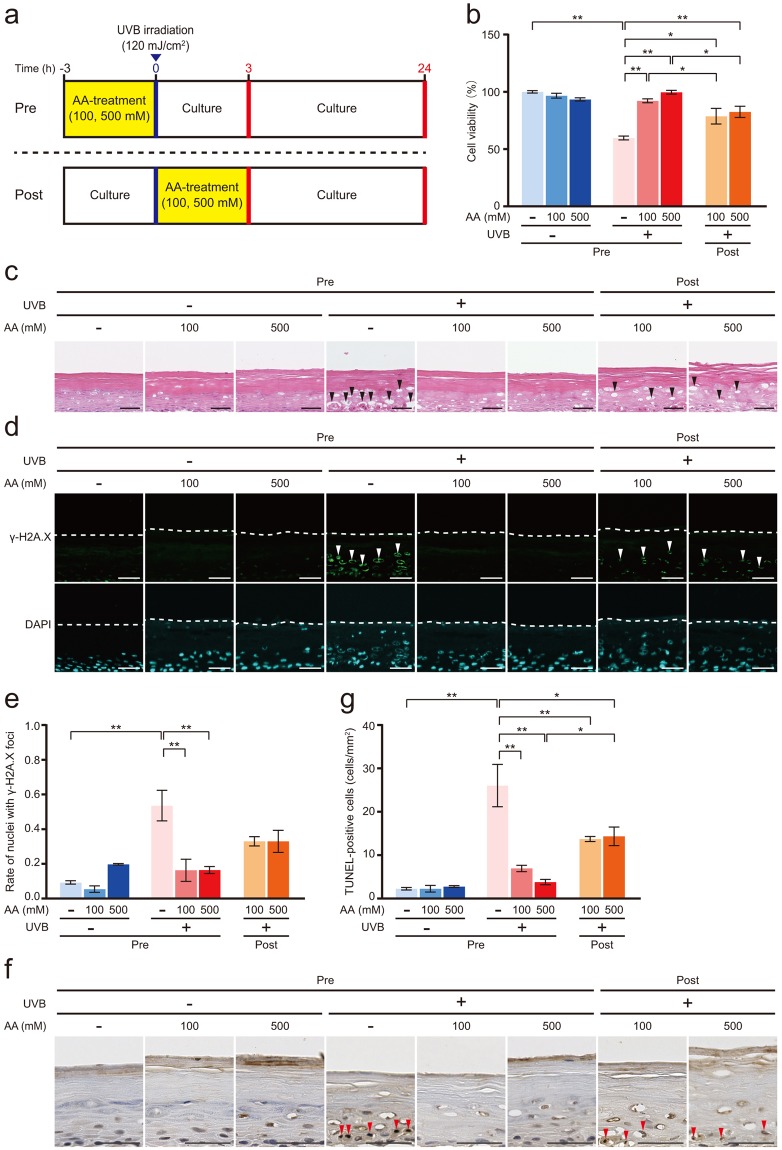
Effect of AA on UVB-induced DNA damage and apoptosis. (**a**) Treatments of 100 and 500 mM AA were applied to the reconstituted human epidermal surface for 3 h before or after 120 mJ/cm^2^ UVB irradiation. After UVB irradiation, the epidermis was cultured for 24 h. (**b**) Cell viability was measured using the MTT assay. Values are presented as the means ± SEM of three wells. (**c**) Histology of the epidermis, as detected by HE staining. Black arrowheads indicate gaps formed by holes. Bar = 50 µm. (**d**) Immunofluorescence staining for γ-H2A.X as a marker of DNA double-strand breaks. The dotted line represents the bottom of the cornified layer. White arrowheads indicate γ-H2A.X-positive nuclei. Bar = 50 µm. (**e**) The percentage of γ-H2A.X-positive nuclei per DAPI-positive nuclei. Values are presented as the means ± SEM of three wells. (**f**) Apoptotic cells were detected by TUNEL staining. Red arrowheads indicate TUNEL-positive cells. Bar = 50 µm. (**g**) Number of TUNEL-positive cells under the cornified layer. Values are presented as the means ± SEM of three wells. The statistical analysis was performed using one-way ANOVA followed by Tukey’s *post hoc* test. *P < 0.05, **P < 0.01. Pre, AA treatment before UVB irradiation. Post, AA treatment after UVB irradiation.

The reconstituted human epidermal surface was treated with 100 or 500 mM AA and cultured for 3 h before (pre-AA treatment) or after (post-AA treatment) 120 mJ/cm^2^ UVB irradiation, and then the epidermis was cultured for 24 h (Fig. 4a). Cell viability of the UVB-irradiated epidermis was significantly reduced by 40% compared with that of the non-irradiated epidermis (Fig. 4b). However, the cell viability of the UVB-irradiated epidermis that had been pre-treated with 100 or 500 mM AA was significantly increased compared with that of the UVB-irradiated epidermis. Significantly higher cell viability was observed in the UVB-irradiated epidermis that had been post-treated with 100 or 500 mM AA than in the UVB-irradiated epidermis. Moreover, significantly higher cell viability was observed in the UVB-irradiated epidermis that had been pre-treated with 100 or 500 mM AA than in the UVB-irradiated epidermis that had been post-treated with 100 or 500 mM AA. The results of the histological analysis are shown in Fig. 4c. Many remarkable gaps formed by holes were observed in the basal, spinous, and granular layers of the UVB-irradiated epidermis. However, the UVB-irradiated epidermis that had been pre-treated with 100 or 500 mM AA was similar to the non-irradiated epidermis. Meanwhile, a few gaps were observed in the UVB-irradiated epidermis that had been post-treated with AA compared with the UVB-irradiated epidermis. Obvious changes in the localization of markers of terminally differentiated keratinocytes, such as citrullinated proteins, filaggrin, loricrin, keratin 10, keratin 14, and collagen type IV, were not observed among the layers of the epidermis (Supplementary Fig. S2). Moreover, significant differences were not detected in levels of the filaggrin, loricrin, keratin 10, keratin 14, and collagen type IV mRNAs (Supplementary Fig. S3). On the other hand, trichohyalin was detected in the spinous, granular, and cornified layers of the UVB-irradiated epidermis and the UVB-irradiated epidermis that had been pre- and post-treated with 100 or 500 mM AA (Supplementary Fig. S2). In addition, significantly higher levels of the trichohyalin mRNA were detected in the UVB-irradiated epidermis than in the non-irradiated epidermis, but a significant difference was not observed between the UVB-irradiated epidermis and the UVB-irradiated epidermis that had been pre- and post-treated with 100 and 500 mM AA (Supplementary Fig. S3). A significantly greater percentage of nuclei displayed γ-H2A.X foci in the UVB-irradiated epidermis than in the non-irradiated epidermis (Fig. 4d,e). However, significantly lower percentages of nuclei displayed γ-H2A.X foci in the UVB-irradiated epidermis that had been pre-treated with 100 and 500 mM AA than in the UVB-irradiated epidermis (Fig. 4e). Similarly, the percentages of nuclei with γ-H2A.X positive foci in the UVB-irradiated epidermis that had been post-treated with 100 and 500 mM AA were lower than in the UVB-irradiated epidermis, but the difference was not significant. A significantly greater number of TUNEL-positive cells was detected in the UVB-irradiated epidermis than in the non-irradiated epidermis (Fig. 4f,g). However, significantly fewer TUNEL-positive cells were observed in the UVB-irradiated epidermis that had been pre-treated with 100 and 500 mM AA than in the UVB-irradiated epidermis. Similarly, significantly fewer TUNEL-positive cells were detected in the UVB-irradiated epidermis that had been post-treated with 100 and 500 mM AA than in the UVB-irradiated epidermis. Moreover, fewer TUNEL-positive cells were observed in the UVB-irradiated epidermis that had been pre-treated with 100 and 500 mM AA than in the UVB-irradiated epidermis that had been post-treated with 100 and 500 mM AA, respectively (Fig. 4g).

### Effect of AA on UVB-induced oxidative damage

**Figure 5:**
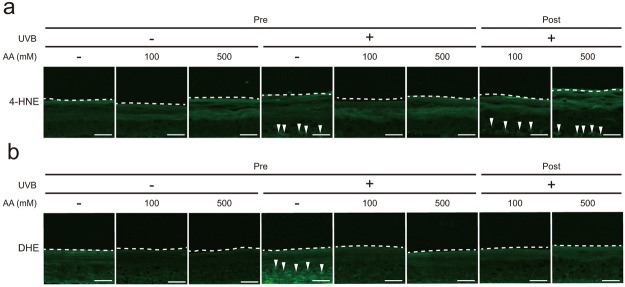
Effects of AA on UVB-induced oxidative damage and ROS production. AA was applied at concentrations of 100 and 500 mM to the reconstituted human epidermal surface for 3 h before or after 120 mJ/cm^2^ UVB irradiation. After UVB irradiation, the epidermis was cultured for 24 h. (**a**) Immunofluorescence staining for 4-HNE as a maker of lipid peroxidation. White arrowheads indicate 4-HNE-positive cells. (**b**) ROS levels in the epidermis were detected by DHE staining. White arrowheads indicate ROS-positive cells. The dotted line represents the bottom of the cornified layer. Bar = 50 µm.

Images of immunofluorescence staining for 4-hydroxynonenal (4-HNE) as a marker of lipid peroxidation are shown in Fig. 5a. Cells displaying 4-HNE-positive staining were observed in the basal, spinous, and granular layers of the UVB-irradiated epidermis. However, 4-HNE staining was not detected in the UVB-irradiated epidermis that had been pre-treated with 100 and 500 mM AA. Moreover, less intense 4-HNE staining was observed in the UVB-irradiated epidermis that had been post-treated with 100 and 500 mM AA than in the UVB-irradiated epidermis (Fig. 5a). Next, we investigated the effect of AA on UVB-induced ROS levels in the epidermis, as detected by dihydroethidium (DHE) staining. Markedly higher DHE fluorescence intensity was detected in the UVB-irradiated epidermis than in the non-irradiated epidermis (Fig. 5b). In addition, DHE fluorescence was detected in the basal, spinous, and granular layers of the UVB-irradiated epidermis. Interestingly, the DHE fluorescence intensity in the UVB-irradiated epidermis that had been pre- and post-treated with 100 and 500 mM was markedly lower than in the UVB-irradiated epidermis.

### Effects of AA on UVB-induced gene expression and tumour necrosis factor-α (TNF-α) release

**Figure 6:**
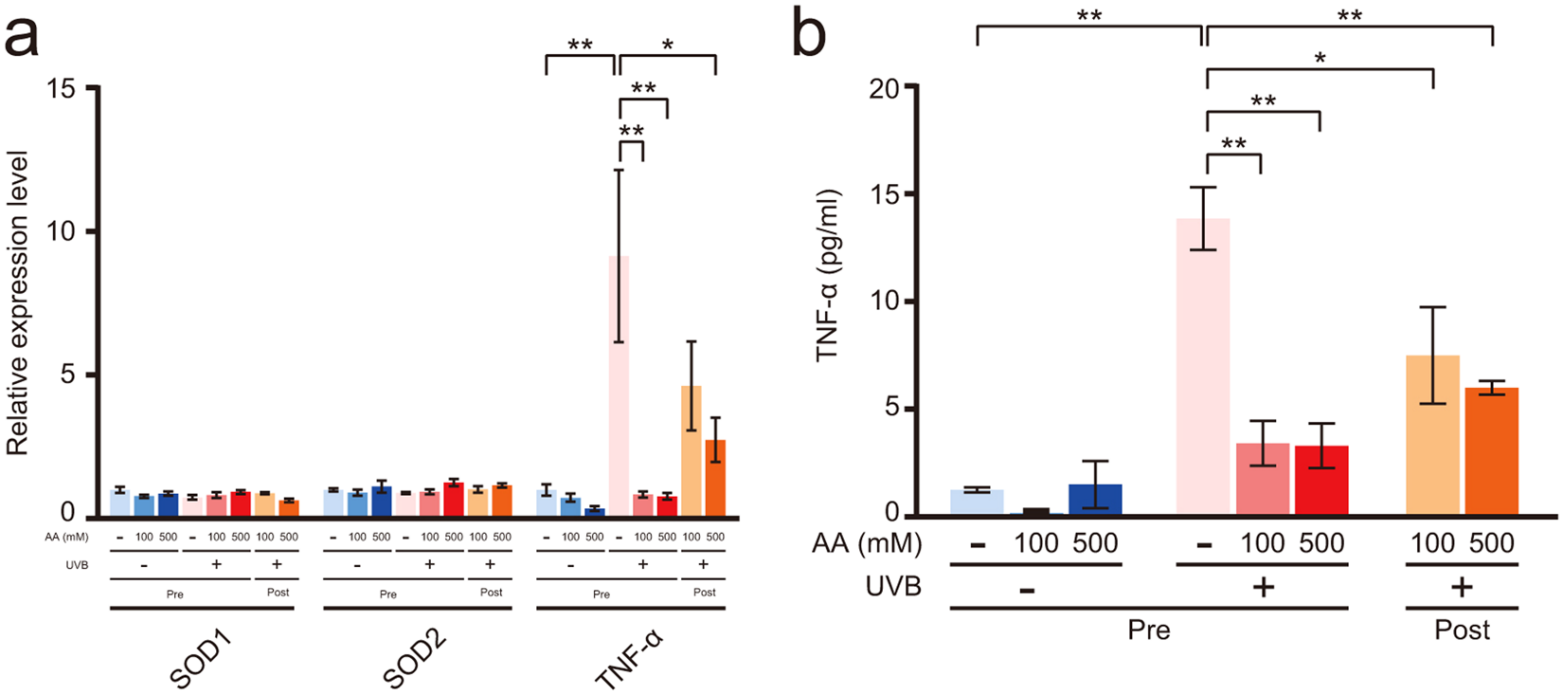
Effects of AA on UVB-induced gene expression and TNF-α production. (**a**) The reconstituted human epidermal surface was treated with 100 and 500 mM AA for 3 h before or after 120 mJ/cm^2^ UVB irradiation. After UVB irradiation, the epidermis was cultured for 3 h. Levels of the SOD1, SOD2, and TNF-α mRNAs were analysed by qPCR. The 18 S rRNA was used as the endogenous control gene. Values are presented as the means ± SEM of three wells. (**b**) After UVB irradiation, the epidermis was cultured for 9 h. The amount of TNF-α in the medium was measured by ELISA. Values are presented as the means ± SEM of three wells. The statistical analysis was performed using one-way ANOVA followed by Tukey’s *post hoc* test. *P < 0.05, **P < 0.01. Pre, AA treatment before UVB irradiation. Post, AA treatment after UVB irradiation.

Levels of the mRNAs encoding the antioxidant enzymes SOD1 and SOD2 and the pro-inflammatory cytokine TNF-α were determined to investigate the effects of AA on UVB-induced gene expression in the epidermis (Fig. 6a). Significant differences in levels of the SOD1 and SOD2 mRNAs were not observed between the non-irradiated and the UVB-irradiated epidermis that had been pre- and post-treated with 100 and 500 mM AA. However, the level of the TNF-α mRNA in the UVB-irradiated epidermis was significantly increased by 9-fold compared with that in the non-irradiated epidermis (Fig. 6a). Moreover, significantly lower levels of the TNF-α mRNA were detected in the UVB-irradiated epidermis that had been pre-treated with 100 and 500 mM AA than in the UVB-irradiated epidermis. Next, we investigated the effect of AA on UVB-induced TNF-α release into the medium. The TNF-α concentration in the medium of the UVB-irradiated epidermis was significantly increased by 11-fold compared with that in the non-irradiated epidermis (Fig. 6b). However, significantly lower TNF-α concentrations were detected in the medium of the UVB-irradiated epidermis that had been pre- and post-treated with 100 and 500 mM AA than in the UVB-irradiated epidermis.

## Discussion

Since little is known about the effect of AA treatments on human skin, we investigated the effect of AA on UVB-induced skin damage using the reconstituted human epidermis. In the present study, AA applied to the epidermal surface was absorbed into the epidermis and prevented UVB-induced skin damage. Interestingly, both pre- and post-AA treatments suppressed UVB-induced skin damage.

The primary function of the epidermis is to protect the internal organs and tissues from environmental factors such as UV irradiation^23^. However, this barrier function results in the inhibition of efficient AA absorption following a topical application. Furthermore, AA mainly exists as a charged molecule at physiological pH, which limits its penetration into the epidermis. Therefore, we first investigated the effects of AA uptake from the apical side of the reconstituted human epidermis, and we detected AA in the epidermis and the medium under the membrane in a dose-dependent manner. Percutaneous absorption of AA increases in a dose-dependent manner, and a maximal absorption of 20% has been achieved in white Yorkshire pigs^24^. We first used 10, 100, 500, and 1000 mM of AA to analyse uptake in the present study. These concentrations are equivalent to 0.2, 2.0, 9.9, and 20% AA, respectively. As 20% AA is the maximal amount absorbed in skin, we used 100 and 500 mM AA to examine the effects of AA on UVB-induced skin damage. Although the differences between the reconstituted human epidermis and pig skin should be considered, our results clearly indicate that AA was absorbed from the apical side of the epidermis and then permeated throughout the reconstituted epidermis.

UVB irradiation has consistently been shown to damage keratinocytes in multiple ways, including by inducing oxidative stress, DNA modification and double-strand breaks^19,20^. However, the harmful effects of UVB irradiation on the reconstituted human epidermis have not been completely elucidated. Therefore, we investigated the harmful effects of UVB irradiation on the reconstituted epidermis. According to the data from the Ministry of the Environment of Japan, 60% of the daily UVB irradiation occurs between 10:00 AM to 2:00 PM^25^. Additionally, the dose of UVB irradiation between 10:00 AM to 2:00 PM is 375 mJ/cm^2^/h. In the present study, the epidermis was irradiated with 50, 80, and 120 mJ/cm^2^ of UVB, doses that correspond to 8, 13, and 20 min of UVB irradiation between 10:00 AM to 2:00 PM in Japan, respectively. Many remarkable gaps formed by holes were observed in the 80 and 120 mJ/cm^2^ UVB-irradiated epidermis. Similar results were obtained from the UVB-irradiated mouse and rat epidermis^26,27^. These studies reported that the histological changes were consistent with intercellular oedema (spongiosis), which is defined as a widening of the intercellular space and a sponge-like appearance of the epidermis^28^. Therefore, the gaps formed by holes were presumed to be caused by intercellular oedema.

Increases in the percentages of TUNEL-positive cells and nuclei with γ-H2A.X foci, as well as enhanced 4-HNE and DHE fluorescence intensity in the UVB-irradiated epidermis were observed in the granular, spinous, and basal layers. However, no changes were observed in the cornified layer. The cornified layer is composed of terminally differentiated, dead keratinocytes^29^. Therefore, the cornified layer must be less susceptible to the influence of UVB irradiation because the nuclei and mitochondria have already been lost.

Trichohyalin is a protein associated with intermediate filaments of the hair follicle^30^. Trichohyalin is only expressed where a hardened keratin structure is needed, such as the inner root sheath of hair follicles, nails, the filiform papillae of the tongue, and the granular and cornified layers of the epidermis^22,31,32^. Although hair follicles, nails, and the epidermis are exposed to UV daily, few studies have focused on the influence of UV on trichohyalin expression. In the present study, trichohyalin was present in the spinous, granular, and cornified layers of the UVB-irradiated reconstituted human epidermis. In addition, markedly higher levels of the trichohyalin mRNA were detected in the UVB-irradiated reconstituted human epidermis than in the non-irradiated epidermis.

Very interestingly, the pre-AA treatment clearly inhibited skin damage, such as cell death, apoptosis, and DNA double-strand breaks, induced by UVB irradiation in the reconstituted human epidermis in the present study. AA has been reported to reduce ROS production induced by UVB irradiation and prevent ROS-mediated cell death and inflammatory cytokine production in cultured epidermal keratinocytes^2,18–20^. However, these studies did not use the reconstituted epidermis model employed in the present study. Moreover, AA suppresses the UVB irradiation-induced reduction in SOD, CAT, GST, and GSH-Px activities in cultured epidermal keratinocytes^33,34^. Thus, a pre-AA treatment may contribute to maintaining the intracellular antioxidant defence system and suppress UVB-induced, ROS-mediated skin damage.

Similarly, the post-AA treatment also inhibited skin damage. However, the viability of cells receiving the post-AA treatment in the UVB-irradiated epidermis was less than cells receiving the pre-AA treatment. Additionally, the number of TUNEL-positive cells, the percentage of nuclei with γ-H2A.X foci, the 4-HNE fluorescence intensity level, and the TNF-α expression level in the post-AA-treated epidermis were greater than the pre-AA-treated epidermis. However, obvious changes in the DHE fluorescence intensity levels were not observed in the UVB-irradiated epidermis between the pre- and post-AA treatment groups. ROS must be produced rapidly in response to UVB irradiation and subsequently react with cellular substrates, resulting in the activation of several signalling cascades^35^. Pre-AA administration, but not oral AA administration, has been shown to rescue cells from acute radiation-induced after X-ray irradiation^36^.

Regarding the pre-AA and post-AA treatments, the cell viability of the pre-AA-treated UVB-irradiated epidermis was greater than the post-AA-treated epidermis. Additionally, the number of TUNEL-positive cells, the percentage of nuclei with γ-H2A.X foci, the 4-HNE fluorescence intensity level, and the TNF-α expression level in the pre-AA-treated epidermis were less than the post-AA-treated epidermis. Thus, the pre-AA treatment is more effective at preventing UVB-induced skin damage than the post-AA treatment.

TNF-α is a primary inflammatory cytokine whose production is induced by UVB in keratinocytes and leads to the progression of an inflammatory cascade^37^. In this study, pre- and post-AA treatments inhibited TNF-α mRNA expression and TNF-α release into the medium. Thus, pre- and post-AA treatments may suppress the UVB-induced inflammatory response in the skin. TNF-α induces keratinocyte apoptosis^38,39^. Therefore, pre- and post-AA treatments must suppress the UVB-induced inflammatory response by downregulating TNF-α mRNA expression and TNF-α release, subsequently protecting the epidermis from UVB irradiation.

In addition, TNF-α promotes collagen degradation in cultured human skin by increasing the levels of matrix metalloproteinase (MMP)-1 and MMP-3 and decreasing procollagen synthesis^40–42^. AA is an essential cofactor for prolyl and lysyl hydroxylases to promote the hydroxylation of proline and lysine to form hydroxyproline and hydroxylysine, respectively^43^. As reported in our previous study, long-term AA treatment increases the levels of type 1 and type 4 collagen mRNAs and type 1 procollagen synthesis in human skin fibroblasts^44^. The major molecular mechanism of skin ageing has been attributed to the loss of mature collagen^45^. Long-term AA treatment of the epidermal surface may suppress UVB-induced photoaging by reducing TNF-α-mediated collagen degradation.

In the present study, we used reconstituted human epidermis as an epidermal equivalent model for evaluation of protective effect of AA on UVB-induced skin damage. However, this model was constituted by normal human epidermal keratinocytes and lacked other components of skin such as fibroblasts, Langerhans cells, melanocytes, and hair follicles. Therefore, further study is needed to elucidate the exact mechanisms of AA treatment using human skin model that containing other skin components.

In conclusion, pre- and post-AA treatments applied to the epidermal surface suppressed UVB-induced cell death, apoptosis, DNA damage, oxidative damage, and ROS and TNF-α production. Therefore, a topical AA treatment of the epidermis prevents UVB-induced skin damage.

## Methods

### Tissue culture

The reconstituted human epidermis (EPI-200, MatTek, Ashland, MA), which is schematically represented in Fig. 1a, was purchased from KURABO (Osaka, Japan). This reconstituted human epidermis is highly differentiated three-dimensional model, which is constituted by normal human epidermal keratinocytes and this model is widely used as epidermal equivalent model. After receipt, the epidermis was removed from the agarose-containing medium and transferred to 6 well culture plates containing a serum-free assay medium (EPI-100-ASY, MatTek) and cultured at 37 °C under 5% CO_2_ in air for 18 to 21 h. In all experiments, the culture was started within 6 h after receipt.

### AA uptake in the epidermis

Fifty µl of 10, 100, 500, or 1000 mM L-AA sodium (Nacalai Tesque, Inc., Kyoto, Japan) and 50 µl of ultrapure water as a control were applied to the epidermal surfaces. After 3 and 6 h, the epidermal surface was washed with phosphate-buffered saline (PBS), and then the epidermis was collected using forceps. The media were also collected and mixed with an equal amount of 10% metaphosphoric acid (MPA) containing 1 mM ethylenediaminetetraacetic acid (EDTA). The epidermis was homogenized with 0.1% sodium dodecyl sulphate using a Minilys homogenizer (Bertin Technologies, Montigny-le-Bretonneux, France) and then mixed with an equal amount of 10% MPA containing 1 mM EDTA. Levels of AA and DHA in the epidermis and the culture media were measured using high-performance liquid chromatography and electrochemical detection, as described previously^46^. AA and DHA contents in the epidermis were normalized to the protein content, which was measured using the Lowry method^47^ with bovine serum albumin (BSA) (Sigma-Aldrich, St. Louis, MO) as a standard.

### UVB irradiation

The epidermis was irradiated with 50, 80, or 120 mJ/cm^2^ of UVB under three UVB lamps (GL20SE, Sankyo, Kanagawa, Japan). For irradiation, the medium was replaced with PBS beneath the culture insert, and the culture plate was irradiated without the plastic plate lid. Control samples were sham-irradiated under the same conditions. The intensity of the UVB radiation was measured using a UV radiometer (UV-340, Custom, Tokyo, Japan) before and after UVB irradiation. After UVB irradiation, the epidermis was cultured for 24 h (Fig. 2a).

### Cell viability assay

Cell viability was measured using a MTT assay kit (Dojindo, Kumamoto, Japan), according to the manufacturer’s protocol. MTT assay is based on the reduction of soluble yellow-colored MTT salt to insoluble purple-colored formazan crystals by mitochondrial dehydrogenase of viable cells. Briefly, the epidermis was washed with PBS and transferred into 24 well culture plate pre-filled with 0.3 ml of MTT solution (final concentration 1 mg/ml). The epidermis was then kept at 37 °C in CO_2_ incubator for 3 h. Subsequently, the epidermis was washed with PBS and transferred into the new 24 well culture plate. The epidermis was then immersed in 2 ml of isopropanol and stored overnight at room temperature in the dark. After extraction, the epidermis was discarded and 0.2 ml of MTT extracts from each well was transferred into a 96 well microtiter plate for reading. The optical density was measured using a wavelength of 570 nm. Cell viability was normalized to the value of the non-irradiated epidermis that was not treated with AA.

### Histology

Frozen epidermal sections (6 μm) were stained with haematoxylin and eosin (HE). Images were obtained using the NanoZoomer 2.0 RS virtual slide scanner (Hamamatsu Photonics, Shizuoka, Japan). The epidermal thickness was measured with a measurement tool of the NDPview2 viewing software (Hamamatsu Photonics).

### Immunohistochemistry

Frozen epidermal sections (6 μm) were fixed with 4% paraformaldehyde for 15 min at 4 °C and incubated with 2% BSA in PBS for 30 min at room temperature to prevent the non-specific binding of antibodies. Sections were then incubated with rabbit anti-keratin 10 (1:1,000 dilution, BioLegend, San Diego, CA), rabbit anti-keratin 14 (1:2,000 dilution, BioLegend), mouse anti-collagen type IV (1:100 dilution, PROGEN, Heidelberg, Germany), rabbit anti-filaggrin (1:5,000 dilution)^48^, mouse anti-torichohyalin (1:500 dilution, Abcam, Cambridge, UK), rabbit anti-loricrin (1:500 dilution, BioLegend), rabbit anti-phospho-histone H2A.X (γ-H2A.X) (1:800 dilution, Cell Signaling Technology, Beverly, MA), and rabbit anti-4-HNE antibodies (1:1,000 dilution, Abcam) for 3 h at room temperature. Sections were then incubated with Alexa Fluor 488-conjugated goat anti-mouse IgG (1:2,500 dilution, Bio-Rad, Hercules, CA) or Alexa Fluor 488-conjugated goat anti-rabbit IgG (1:2,500 dilution, Bio-Rad), and 4,6-diamidino-phenyl indole dihydrochloride (DAPI) (1:5,000, Sigma-Aldrich) for 1 h at room temperature. The fluorescence signals (Excitation 488 nm/Emission 519 nm) were detected with the FLUOVIEW FV10i laser scanning confocal microscope (OYMPUS, Tokyo, Japan). The percentages of γ-H2A.X-positive nuclei per DAPI-positive nuclei were calculated in 3 randomly selected 0.04 mm^2^ fields (200 µm × 200 µm) from each section.

### Detection of citrullinated proteins

Frozen epidermal sections (6 μm) were fixed with 4% paraformaldehyde for 15 min at 4 °C and incubated with 2% normal goat serum in PBS for 30 min at room temperature. Citrullinated proteins were detected using methods described by Senshu *et al*.^49^. Briefly, citrulline residues on the sections were modified in modification medium (1 volume of 1% diacetyl monoxime/0.5% antipyrine/1 N acetic acid and 2 volumes of 20% H_3_PO_4_/25% H_2_SO_4_/0.025% FeCl_3_) for 3 h at 37 °C. Sections were incubated overnight with mouse anti-modified citrulline (1:1,500 dilution)^49^ at room temperature. Sections were then incubated with Alexa Fluor 488-conjugated goat anti-mouse IgG (1:2500 dilution) for 1 h at room temperature. Fluorescence signals were detected with the FLUOVIEW FV10i laser scanning confocal microscope.

### DHE staining

DHE (Wako Pure Chemical, Osaka, Japan), an fluorescent dye that detects oxidative stress, was used to detect superoxide anion radicals in frozen skin tissues as previously described^50^. Frozen epidermal sections (6 µm) were incubated with 5 µM DHE at 37 °C for 30 min in a light-protected chamber. The fluorescence signals were detected with the FLUOVIEW FV10i laser scanning confocal microscope.

### Apoptosis assay

Apoptotic cells were detected by terminal deoxynucleotidyl transferase-mediated dUTP-biotin nick end labelling (TUNEL) using the DeadEnd™ Colorimetric TUNEL System (Promega, Madison, WI), according to the manufacturer’s protocol. Images were obtained using the NanoZoomer 2.0 RS virtual slide scanner (Hamamatsu photonics). The number of TUNEL-positive cells under the cornified layer were counted in 5 randomly selected 1 mm^2^ square fields from each section.

### Extraction of total RNA and cDNA synthesis

Total RNA was extracted using ISOGEN (Wako Pure Chemical)^51^. RNA concentrations were determined and confirmed as free from protein contamination by measuring the absorbance at 260 and 280 nm. Then, cDNAs were synthesized using SuperScript III reverse transcriptase (Invitrogen, Carlsbad, CA) according to the manufacturer’s protocol. The cDNA samples were stored at −80 °C until use.

### Quantitative polymerase chain reaction (qPCR)

Using the THUNDERBIRD^®^ SYBR qPCR Mix (TOYOBO, Osaka, Japan), qPCR was performed according to the manufacturer’s protocol. The primers for SOD1 and SOD2, TNF‐α, and 18 S ribosomal RNA (18 S rRNA) were purchased from Integrated DNA Technologies, Inc. (Coralville, IA). The primer sequences are provided in Supplemental Table 1 S. The reactions were performed using the real-time PCR equipment (StepOne Plus, Applied Biosystems, Foster City, CA). The amplification protocol consisted of denaturation at 95 °C for 1 min and 40 cycles of 95 °C for 15 s and 60 °C for 1 min. A standard curve was designed for the quantitative analysis of the level of each mRNA; in this case, an aliquot from each experimental sample was used to generate standard curves. The relative expression levels of each gene were normalized to the 18 S rRNA. The expression levels observed in the non-irradiated epidermis without AA were assigned a relative value of 1.

### Determination of TNF-α levels

TNF-α levels were measured using an enzyme-linked immunosorbent assay (ELISA) kit (R&D Systems, Minneapolis, MN), according to the manufacturer’s instructions. TNF-α levels were calculated based on the standard curve and expressed in pg/ml.

### Statistical analysis

Results are presented as means ± standard errors of the mean (SEM). The significance of statistical differences between experimental groups was determined using one-way analysis of variance (ANOVA) followed by Dunnett’s or Tukey’s *post hoc* tests with Graph Pad Prism 6 software (GraphPad Software Inc., San Diego, CA). Differences were considered statistically significant at *P* < 0.05.

## Electronic supplementary material

Supplementary information
