# Supplementary material for: Protective effect of pre- and post-vitamin C treatments on UVB-irradiation-induced skin damage
Source: Sci Rep. 2018 Nov 1;8:16199. doi: 10.1038/s41598-018-34530-4 (PMC6212420; doi:10.1038/s41598-018-34530-4)
Supplement: Supplementary file 1 — Supplementary information [file 41598_2018_34530_MOESM1_ESM.pdf]

Protective effect of pre- and post-vitamin C treatments on UVB-irradiation-induced skin damage

Saki Kawashima [1,2,3], Tomoko Funakoshi [1], Yasunori Sato [4], Norikatsu Saito [5], Hajime Ohsawa [5], Katsumi Kurita [5], Kisaburo Nagata [3], Masayuki Yoshida [2], and Akihito Ishigami [1,*]

[1] Molecular Regulation of Aging, Tokyo Metropolitan Institute of Gerontology, Tokyo 173-0015, Japan

[2] Department of Life Science and Bioethics, Graduate School of Medicine, Tokyo Medical and Dental University, Tokyo 113-8510, Japan

[3] Department of Biomolecular Science, Faculty of Science, Toho University, Chiba 274-8510, Japan

[4] Department of Bioenvironmental Pharmacy, Faculty of Pharmaceutical Sciences, Hokuriku University, Ishikawa 920-1181, Japan

[5] Risou Co., Ltd., Tokyo 104-0061, Japan

*** Corresponding author:** Akihito Ishigami, Ph.D., Molecular Regulation of Aging, Tokyo Metropolitan Institute of Gerontology (TMIG), 35-2 Sakae-cho, Itabashi-ku, Tokyo 173-0015, Japan. Phone +81-3-3964-3241, E-mail: ishigami@tmig.or.jp

Supplemental Methods

qPCR. Using the THUNDERBIRD® TaqMan qPCR mix (TOYOBO), qPCR was performed according to the manufacturer's protocol. The primers and 5′-carboxyfluorescein (6-FAM)/N, Ndiethyl-4-(4-nitronaphthalen-1-ylazo)-phenylamine (ZEN)/Iowa Black® FQ-3′ double-quenched probes for filaggrin, loricrin, keratin 10, keratin 14, collagen type IV, trichohyalin, and 18S rRNA were purchased from Integrated DNA Technologies, Inc. The primer sequences are provided in Supplemental Table 1S. The reactions were performed using real-time PCR equipment (StepOne Plus, Applied Biosystems). The amplification protocol consisted of denaturation at 95 °C for 1 min and 40 cycles of 95 °C for 15 s and 60 °C for 1 min. A standard curve method was designed for the quantitative analysis of the expression of each mRNA; in this case, an aliquot from each experimental sample was used to generate standard curves. The relative expression levels of each gene were normalized to the 18S rRNA. The expression levels in the non-irradiated epidermis without AA were assigned a relative value of 1.

Supplemental Table S1. Primer sets used for the qPCR analysis.

Target gene		Sequence
SOD1	Forward	5'-ACTGGTGGTCCATGAAAAAGC-3'
	Reverse	5'-AACGACTTCCAGCGTTTCCT-3'
SOD2	Forward	5'-GCCTACGTGAACAACCTGAAC-3'
	Reverse	5'-TGAGGTTTGTCCAGAAAATGC-3'
TNF-α	Forward	5'-TCAGCTTGAGGGTTTGCTAC-3'
	Reverse	5'-TGCACTTTGGAGTGATCGG-3'
18S rRNA (for SYBR)	Forward	5'-GGACATCTAAGGGCATCACAG-3'
	Reverse	5'-GAGACTCTGGCATGCTAACTAG-3'
Filaggrin	Forward	5'-GCCATGTCTCCAAACTAAACC-3'
	Reverse	5'-GCAGTCCTCACAGTTCTAGT-3'
	Probe	/56-FAM/TGACCTTTT/ZEN/TGCCTTTCAGTGCCC /3IABkFQ/
Loricrin	Forward	5'-GTCTTCACGCAGTCCACT-3'
	Reverse	5'-TCTCCTCACTCACCCTTCC-3'
	Probe	/56-FAM/TATCAGAAA/ZEN/AAGCAGCCCACCCCT/3IABkFQ/
Keratin 10	Forward	5'-GCAGAGCTACCTCATTCTCATAC-3'
	Reverse	5'-ATCGATGACCTTAAAAATCAGATTCTC-3'
	Probe	/56-FAM/TCCTGCTTC/ZEN/AGATCGACAATGCCAG/3IABkFQ/
Keratin 14	Forward	5'-ACACCACCTTGCCATCG-3'
	Reverse	5'-CCTCCTCCCAGTTCTCCT-3'
	Probe	/56-FAM/ACCTCCTCC/ZEN/AGCCGCCAAATC/3IABkFQ/
Collagen type IV	Forward	5'-TGAGTCAGGCTTCATTATGTTCT-3'
	Reverse	5'-AGAGAGGAGCGAGATGTTCA-3'
	Probe	/56-FAM/TCATACAGA/ZEN/CTTGGCAGCGGCT/3IABkFQ/
Trichohyalin	Forward	5'-TGATGTCACAGATGCTTCTCAG-3'
	Reverse	5'-ACCCAGTACACTTGCCTGT-3'
	Probe	/56-FAM/AGTTCAAGT/ZEN/AAACCCACTTCACCAGAGG/3IABkFQ/
18S rRNA (for TaqMan)	Forward	5'-GGACATCTAAGGGCATCACAG-3'
	Reverse	5'-GAGACTCTGGCATGCTAACTAG-3'
	Probe	/56-FAM/TGCTCAATC/ZEN/TCGGGTGGCTGAA/3IABkFQ/

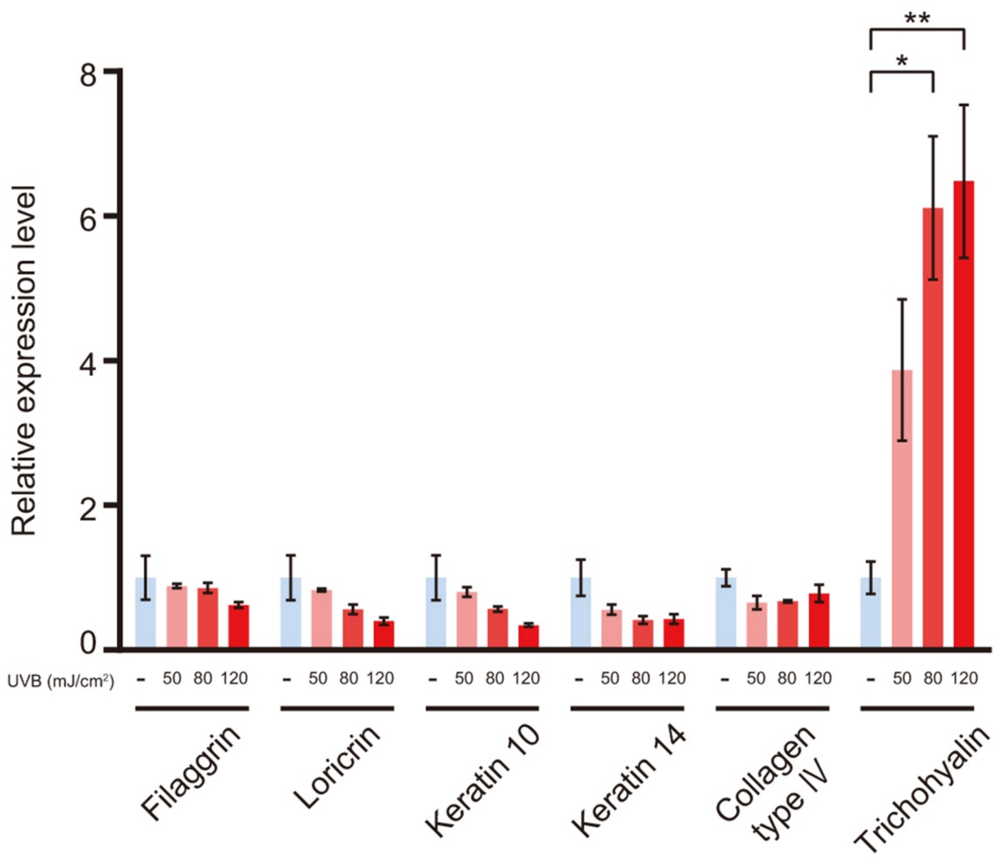

Supplemental Figure S1. UVB induced gene expression in the reconstituted human epidermis. The reconstituted human epidermis was irradiated with 50, 80 or 120 mJ/cm^2 UVB. After UVB irradiation, the epidermis was cultured for 3 h. Levels of the filaggrin, loricrin, keratin 10, keratin 14, collagen type IV, and trichohyalin mRNAs were analysed by qPCR. The 18S rRNA was used as the endogenous control gene. Values are presented as the means ± SEM of three wells. The statistical analysis was performed using one-way ANOVA followed by Tukey's *post hoc* test. *$P < 0.05$, **$P < 0.01$.

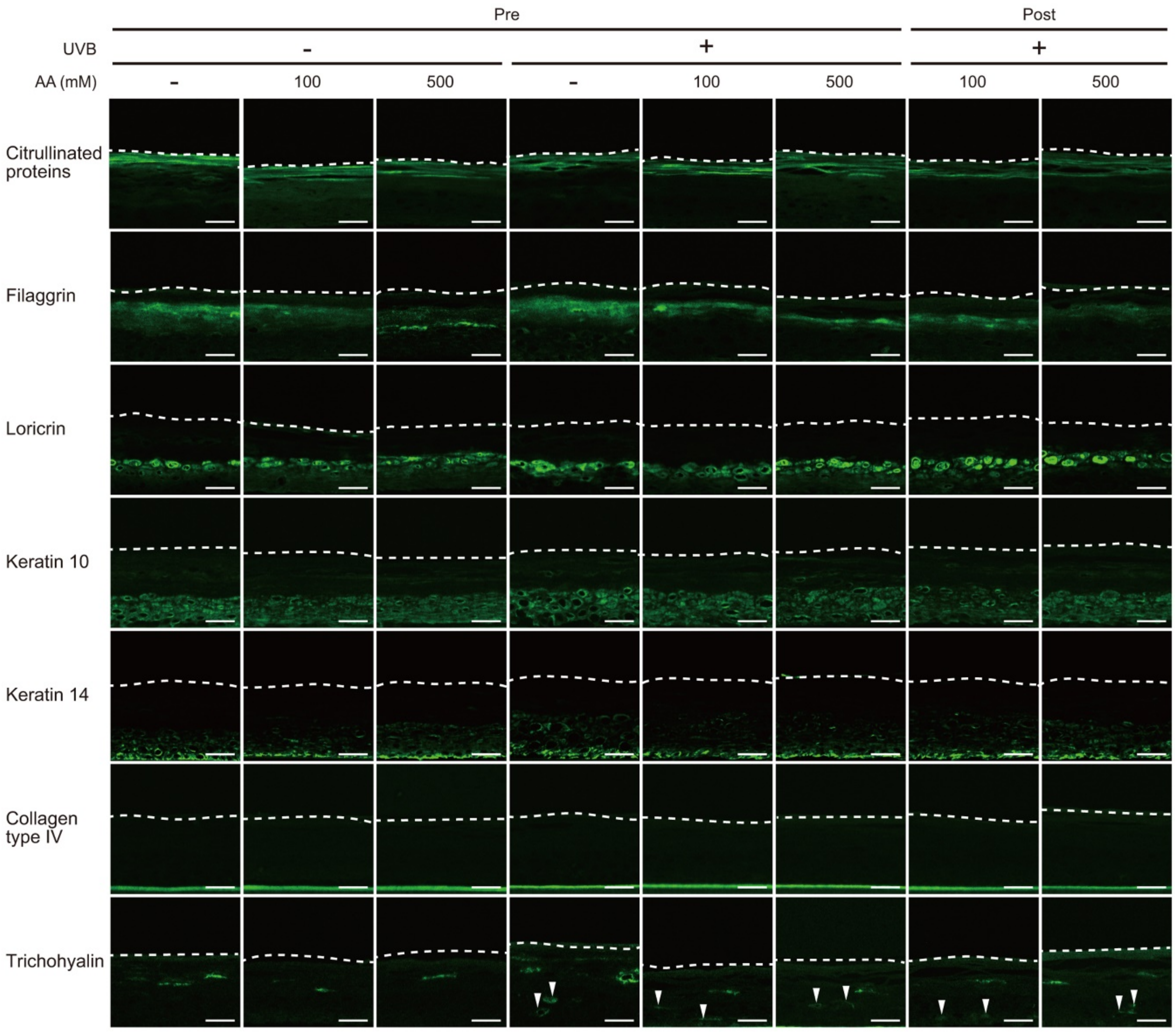

Supplemental Figure S2. Immunofluorescence staining for citrullinated proteins, filaggrin, loricrin, keratin 10, keratin 14, collagen type IV, and trichohyalin in the UVB-irradiated epidermis that had been pre- and post-treated with 100 and 500 mM AA. The dotted line represents the bottom of the cornified layer. White arrowheads indicate trichohyalin. Bar = 50 μm. Pre, AA treatment before UVB irradiation. Post, AA treatment after UVB irradiation.

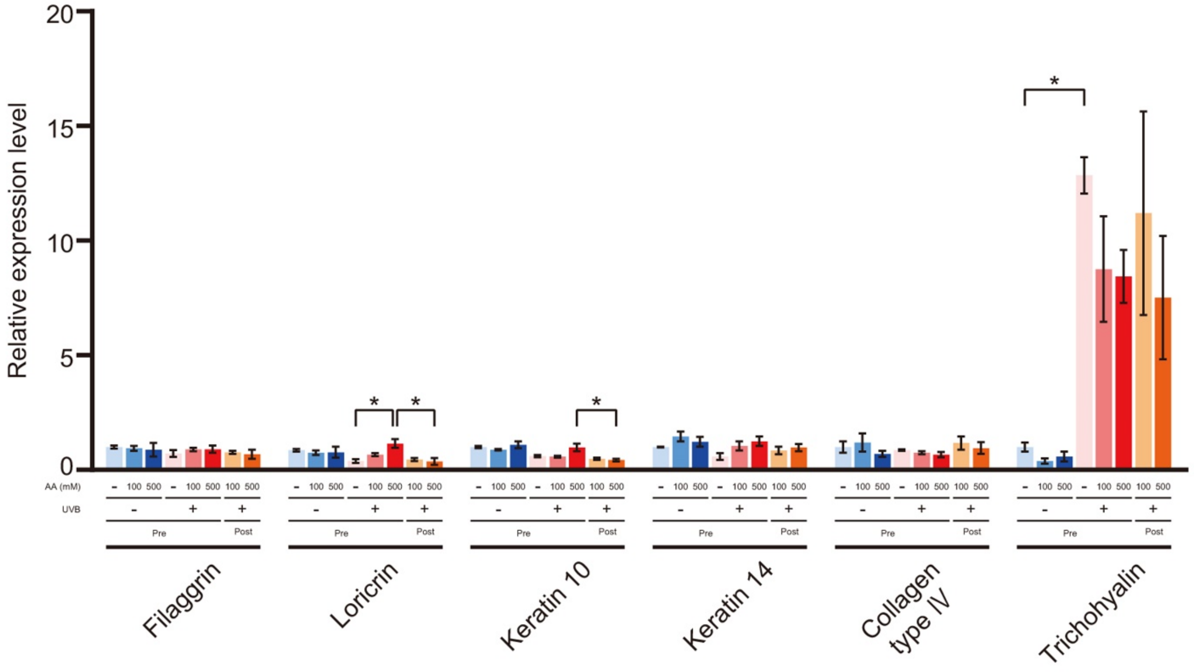

Supplemental Figure S3. Effect of AA on UVB-induced gene expression. Treatments of 100 and 500 mM AA were applied to the reconstituted human epidermal surface for 3 h before or after 120 mJ/cm^2 UVB irradiation. After UVB irradiation, the epidermis was cultured for 3 h. Levels of the filaggrin, loricrin, keratin 10, keratin 14, collagen type IV, and trichohyalin mRNAs were analysed by qPCR. The 18S rRNA was used as the endogenous control gene. Values are presented as the means ± SEM of three wells. The statistical analysis was performed using one-way ANOVA followed by Tukey's *post hoc* test. Pre, AA treatment before UVB irradiation. Post, AA treatment after UVB irradiation. *$P < 0.05$.
